# Deprescribing process in demented patients: What is the rationale?

**DOI:** 10.1192/j.eurpsy.2021.184

**Published:** 2021-08-13

**Authors:** G. Stoppe

**Affiliations:** Practice-counsel-research, MentAge, Basel, Switzerland

**Keywords:** Deprescribing, dementia, polypharmacy, delirium

## Abstract

Polypharmacy is rather a rule than an exemption in the elderly. This applies also to the demented population, whether they live in private homes or in nursing homes. The application of multiple drugs increases the risk to develop delirium, to promote falling and to hasten cognitive decline, What can be done to reduce these risks? First of all, drugs should be given on the basis of an appropriate assessment. Pain e.g. may be misunderstood as challenging bevhaviour. Side affects might be misunderstood as newly occuring symptoms. Drugs should be prescribed with a written protocol, what the drug is expected to do. If this does not occur, the drug should be deprescribed. In addition, antidepressants should be deprescribed. Many demented patients receive more than two of them, mostly for years. Depresciption follows the evidence, that antidepressants are not much helpful in dementia. They may induce hyponatriamia, too. The deprescription of benzodiazepines requires patience and a long tapering-out. And overall, what about the antipsychotics? They shall be given at a minimum dosage and duration. That means, that drug pauses should be established regularly. And finally, what about the antibiotics, antihypertensive drugs and more? Having in mind, that severe dementia is mostly a state, where the priniciples of palliative medicine should be applied, also many of these drugs can be deprescribed.

**Disclosure:**

No significant relationships.

